# Daily physical activity patterns across the menstrual cycle in eumenorrheic female athletes: an accelerometry-based study

**DOI:** 10.3389/fspor.2026.1793624

**Published:** 2026-07-20

**Authors:** Paula Recacha-Ponce, Carla Hernando, Ana Capdevila-Seder, Eladio Collado-Boira, María-Pilar Suárez-Alcázar, Pablo Salas Medina, Carlos Hernando

**Affiliations:** 1 Facultat de Ciencies de la Salut, Universitat Jaume I, Castellón de la Plana, Spain; 2 Department of Mathematics. Universitat Jaume I, Castelló de la Plana, Spain; 3 Departament of Educaction and Specifis Diactics, Universitat Jaume I, Castelló de la Plana, Spain

**Keywords:** accelerometry, energy consumption, eumenorrheic women, female athletes, hormonal fluctuations, menstrual cycle, physical activity

## Abstract

**Background:**

Hormonal fluctuations during the menstrual cycle (MC) influence various physiological parameters, yet their impact on daily physical activity (PA) behavior in naturally menstruating women remains under-researched, often lacking rigorous hormonal verification.

**Methods:**

This prospective observational study aimed to examine variations in daily PA intensity and energy consumption across MC phases in eumenorrheic, female athletes [classification as level II or level III athletes according to McKay et al. (2022)] using accelerometry as an objective tool to measure PA. Twenty female athletes (26.6 ± 5.9 years) with regular cycles were monitored using wrist-worn GENEActiv accelerometers over one complete natural cycle. MC phases were classified into seven stages (early, mid, and late follicular; ovulatory; early, mid, and late luteal) using a combination of self-reported bleeding, daily urinary luteinizing hormone (LH) testing, and serum hormone analysis (progesterone >16 nmol·L⁻^1^ to confirm ovulation). PA intensity was categorized into six levels (sedentary to extremely vigorous) using validated triaxial cut-points, and energy consumption (kcal·kg^−1^·min^−1^) was estimated from accelerometry data.

**Results:**

No significant differences were found across MC phases for sedentary, light, or moderate PA *p* > 0.05. However, time spent in vigorous, very vigorous, and extremely vigorous intensities showed significant phase-dependent variations *p* = 0.004; 0.001 y 0.001 respectively, with the lowest values consistently recorded during the ovulatory phase. Energy consumption also reached its minimum during ovulation *p* = 0.009, while total wear time remained stable.

**Conclusion:**

In eumenorrheic athletes, the MC does not substantially modify overall daily activity volume or lower-intensity behaviors. However, high-intensity activity and energy consumption are sensitive to cyclical hormonal fluctuations, specifically decreasing during the ovulatory phase. These findings suggest that higher-intensity activity domains may be more susceptible to menstrual cycle fluctuations than overall activity volume. Therefore, training prescription and load monitoring should consider individual cycle patterns and hormonal profiles rather than assuming behavioral stability across all intensity levels.

## 1 Introduction

The menstrual cycle (MC) is characterized by cyclic fluctuations in the concentrations of steroid hormones, particularly estrogen and progesterone. There are studies conﬁrming that hormones related to the MC inﬂuence cardiovascular, respiratory, neuromuscular, neurocognitive, and metabolic parameters, and consequently, physical ﬁtness (1–4). In the same way, research supports the presence of symptomatology associated with the MC (5, 6). These changes are more pronounced during the early follicular and late luteal phases, where symptoms are exacerbated. Conversely, the ovulatory and mid-luteal phases are characterized by more stable hormonal profiles, which may promote symptom stability (7). Physical activity (PA) is commonly defined as any bodily movement produced by skeletal muscles that results in energy consumption, encompassing activities across occupational, sports, household, and other domains (8).

This could lead to the hypothesis that the daily PA of women with natural MC is also affected as a consequence of these fluctuations. Currently, there is no research addressing this topic in relation to the PA of women with regular natural MC. Beyond this, numerous studies on the MC have been conducted without verifying the hormonal patterns that confirm whether a MC is eumenorrheic, highlighting the need for precise hormonal profiling (9, 10). A recent systematic review restricted to studies with high methodological standards confirms that findings remain highly heterogeneous and limited by small sample sizes, a lack of elite athlete representation, and a moderate-to-high risk of bias (11).This is particularly relevant given the physiological demands of athletic training and the prevalence of ovulatory disturbances in this population (12). Without verifying hormonal patterns, conclusions may be biased by generalizing findings across ovulatory women and those with deficient luteal phases or anovulatory cycles, where typical physiological hormonal fluctuations are absent (12). Likewise, identifying literature that accurately classifies the stages of the MC is a challenging task, as studies to date have employed varied nomenclatures without standardizing the phases of the MC to enable comparative analyses and draw reliable conclusions (13).

Objective monitoring of daily PA through accelerometers provides a robust method to quantify movement patterns and energy consumption across the MC (14–17). These tools offer a more reliable assessment compared to self-reported activity, which may be subject to recall bias (18). Understanding whether PA levels increase or decrease during specific cycle phases in eumenorrheic women could inform tailored training and recovery strategies. Some studies indicate that female athletes may maintain consistent activity levels across all phases, possibly due to training regimens or psychological resilience (19). However, despite adherence to training discipline, the MC may still influence their daily routines and overall PA levels, indirectly impacting their quality of life. Nevertheless, while the influence of the MC on athletic performance has gained attention in recent research (19–21), the variation in daily PA across the MC remains underexplored. Implementing objective monitoring of physical activity, combined with accurate verification of eumenorrheic MC, could provide valuable insights into this understudied area. Specifically, for female athletes, understanding the interaction between MC phases and PA enables the development of personalized training protocols, thereby optimizing both preparation and health.

This study aims to examine variations in daily physical activity levels and intensities across the seven phases of the menstrual cycle in eumenorrheic athletes. Additionally, it seeks to explore how these activity patterns relate to hormonal fluctuations throughout the cycle, providing a basis for more personalized training and health strategies for female athletes.

## 2 Materials and methods

### 2.1 Ethical considerations

The study was conducted in accordance with the Declaration of Helsinki and approved by the local Ethics Committee of Jaume I University (CD/77/2020). The trial was prospectively registered at ClinicalTrials.gov (ID: NCT05576740). All participants provided written informed consent before any study procedures.

### 2.2 Consent to participate

All participants provided written informed consent prior to enrollment. Participants were informed of the study's objectives, procedures, and their right to withdraw at any time.

### 2.3 Participants

A total of 180 women were initially screened for eligibility through social media and local sports networks. Of these, 153 were excluded: 95 did not meet the inclusion criteria for the present study, 44 declined to participate, and 14 were allocated to a different study arm not reported here (users of hormonal contraceptives). Consequently, 27 physically active women with self-reported regular natural menstrual cycles were initially enrolled.

Inclusion criteria were: i) female sex; ii) age between 18 and 40 years; iii) classification as level II or level III athletes according to McKay et al. (22); iv) regular natural menstrual cycles (21–35 days) for at least the previous three months; and v) no use of hormonal contraceptives during the previous three months. Exclusion criteria were: i) refusal to participate; ii) pregnancy or breastfeeding; iii) any chronic disease, metabolic disorder, or medical condition known to affect endocrine function; and iv) regular use of medications or dietary supplements that could influence hormonal status, metabolism, or physical activity behaviour.

**Figure 1:**
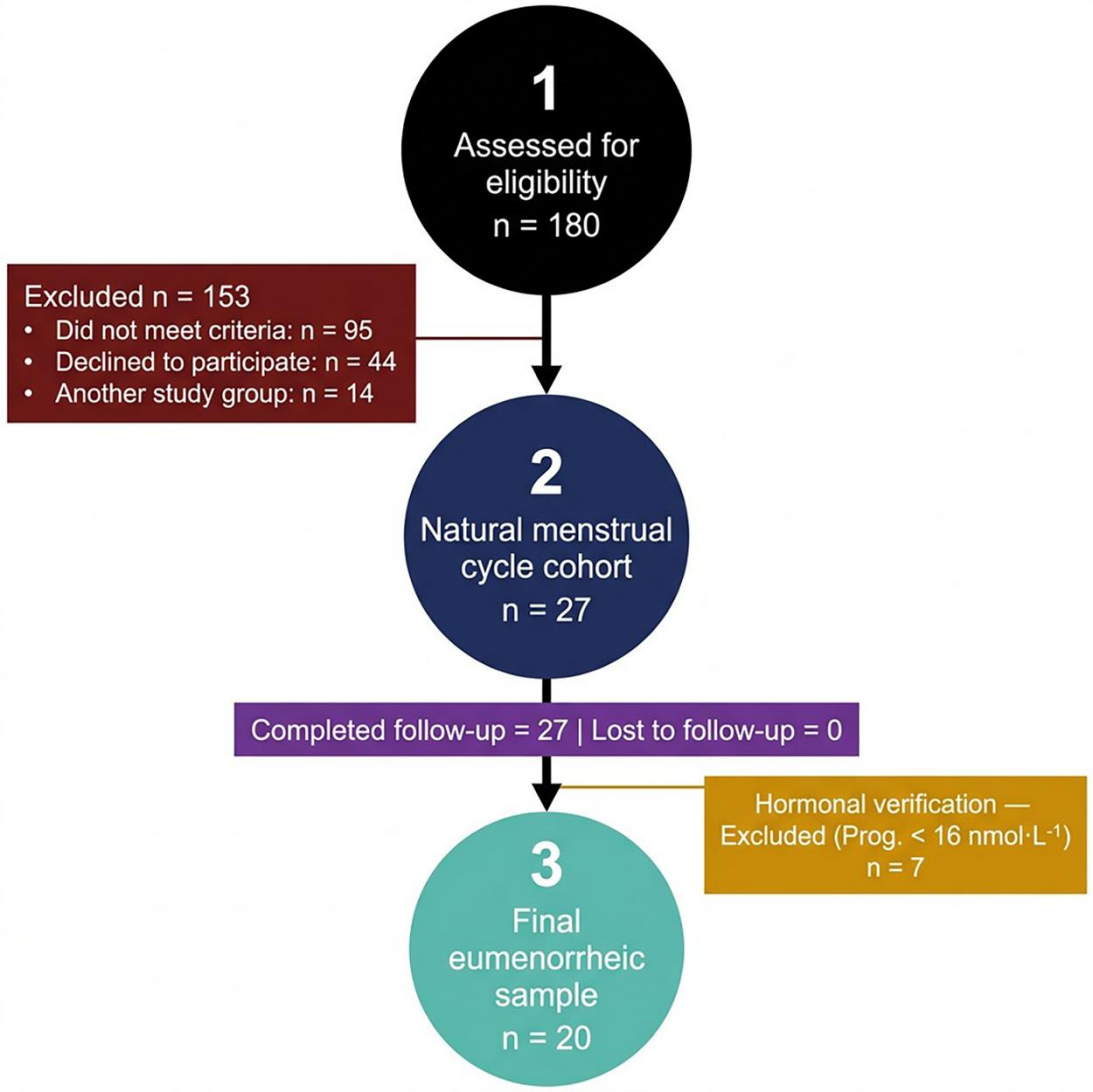
Flow diagram of participant recruitment, eligibility assessment, hormonal verification, and final inclusion in the analysis.

All 27 participants completed the entire monitoring period. However, following objective hormonal verification, 7 women were excluded from the final analysis because their serum progesterone concentration during the mid-luteal phase was below the predefined threshold of 16 nmol·L⁻^1^, indicating anovulatory cycles or luteal phase deficiency. In addition, individual LH and FSH profiles were examined to confirm physiological hormonal patterns throughout the menstrual cycle. Therefore, the final sample consisted of 20 eumenorrheic female athletes (26.6 ± 5.9 years). The participant flow through recruitment, eligibility assessment, hormonal verification, and final inclusion is shown in Figure 1. Sociodemographic characteristics are summarized in Table 1.

**Table 1:** Participant characteristics.

Measurement	(*n* = 20)
Age (years)	26.55 ± 5.88
Height (cm)	165.21 ± 6.52
Weight (kg)	63.25 ± 10.05
BMI (kg·m^−2^)	23.05 ± 2.43
Years practicing sports	13.75 ± 8.22
Training minutes (min·week^−1^)	451 ± 201.83
Cycle duration (days)*	27.90 ± 2.73
Menstrual bleeding duration (days)	4.41 ± 1.03

Values are presented as mean ± SD.

* Cycle duration: defined as days from day 1 of bleeding until the next bleeding. Body composition values correspond to the first assessment (EFP).

### 2.4 Experimental design

This prospective study with a cross-sectional approach assessed PA across a full MC. All participants completed three laboratory visits and underwent continuous monitoring of daily physical activity.

Three laboratory visits were scheduled, coinciding with the early follicular phase, ovulation (24–48 h after a positive LH test), and the mid-luteal phase (7 days post-positive LH test). At each of these visits, blood samples were collected for endocrine analysis. Prior to blood collection, a structured anamnesis verified the athlete's physiologically stable health status, confirming the absence of acute illness or medication use in the previous 48 h. Additionally, participants confirmed compliance with pre-test requirements: a minimum of 7 h of sleep, a two-hour fasting period, and avoidance of strenuous physical activity for at least 24 h. Finally, anthropometric measurements, including body weight and BMI, were performed using a bioelectrical impedance scale (Tanita BC-780MA, Tanita Corp., Tokyo, Japan) (23).

These measurements adhered to the manufacturer's guidelines, involving the cleaning of the contact plate with a disinfectant gel before bioimpedance testing, without applying conductive gel to the feet. Athletes were assessed wearing light sports clothing, without shoes, and after a two-hour fasting period. Body weight, measured via bioelectrical impedance, was also used to adjust accelerometer settings at each visit. Height was measured only during the first visit using a SECA 213 portable stadiometer (Seca GmbH & Co. Kg, Hamburg, Germany). In parallel, daily PA was continuously monitored 24 h a day throughout the entire MC using GENEActiv wrist-worn accelerometers (8, 24). To ensure seamless data collection and capture all PA performed by the athletes, devices were exchanged at each laboratory visit, providing a complete record of the entire menstrual cycle.

#### 2.4.1 Hormonal phase determination and verification

The MC phases were determined through a combination of: i) self-reported menstrual bleeding onset (defining day 1 of the cycle), ii) daily urinary luteinizing hormone (LH) testing starting from cycle day 8 until a positive result was obtained, and iii) serum hormone analysis (progesterone >16 nmol·L⁻^1^ in the mid-luteal phase confirming ovulation). If any of these criteria were not met, participants were excluded.

In accordance with the framework established by Elliott-Sale et al. (13, 25) and the methodological adaptation described by Recacha-Ponce et al. (6), the MC was divided into seven distinct phases. Phase duration and timing were retrospectively adjusted for each participant based on menstrual bleeding onset and the urinary LH surge, as follows:

- **Early follicular phase (EFP):** Defined as the period from the onset of menstrual bleeding to cycle day 5.
- **Midfollicular phase (MFP):** Defined as the interval between the early follicular phase and the late follicular phase; therefore, its duration was individualized according to each participant's cycle length.
- **Late follicular phase (LFP):** Defined as the 24-hour period immediately preceding the luteinizing hormone (LH) surge.
- **Ovulatory phase (Ov):** Identified by a positive urinary LH test. In the present study, this phase was operationally defined as the 24 h following the positive LH test.
- **Early luteal phase (ELP):** Defined as the interval from the end of the ovulatory phase to the beginning of the midluteal phase. In the present study, this phase lasted 3 days.
- **Midluteal phase (MLP):** Defined as the 3-day window occurring 7 ± 1 days after the positive LH test.
- **Late luteal phase (LLP):** Defined as the interval from the end of the midluteal phase to the onset of the subsequent menstrual bleeding; its duration was individualized for each participant.

### 2.5 Study protocol

Participants were instructed to maintain their usual diet and training routines during the study period. To control for factors influencing hormonal measurements and performance, assessments were standardized (26, 27). Visits were scheduled at consistent times of day to account for circadian variation (28); Laboratory conditions (temperature, humidity) were controlled.

#### 2.5.1 Physical activity monitoring

Daily PA was assessed continuously using GENEActiv accelerometers (Activinsights Ltd., Kimbolton, Cambridgeshire, UK) worn on the non-dominant wrist (29, 30). The accelerometers and their output have been previously validated by Esliger DW et al. (17). The devices recorded triaxial acceleration data, and categorization of activity intensity into six levels (sedentary, light, moderate, vigorous, very vigorous, extremely vigorous) following the methodology of (29). The energy consumption of each subject was estimated based on this distribution of intensities. For this purpose, one MET was assumed to represent 1 kcal·kg^−1^·h^−1^ (31–33), and the methodology used by Hernando et al. 2018 was adopted (29).

Accelerometers were worn on the non-dominant wrist to avoid recording everyday movements performed only with the arm. Furthermore, precise instructions were given on the importance of not removing them at any time of day or night, including underwater use, thus recording PA data 24 h a day. The accelerometers were replaced weekly to prevent memory saturation and battery depletion, ensuring uninterrupted monitoring throughout all phases of the menstrual cycle. Subsequently, energy consumption was normalized according to body weight (29).

To control for the influence of competitive stress and peak training loads, all monitoring was conducted during the off-season period (summer season).

#### 2.5.2 Blood analysis

A venous blood specimen was obtained from the antecubital vein. After centrifugation, serum was transported to the laboratory for immediate analysis. All blood draws were performed before the physical testing. Serum measurements were carried out on an Architect c-8000 analyzer (Abbott Laboratories, Abbott Park, IL, USA) using chemiluminescent assays to determine luteinizing hormone (LH), follicle-stimulating hormone (FSH), 17*β*-estradiol and progesterone.

### 2.6 Statistical analysis

Statistical analyses were conducted using the Statistical Package for the Social Sciences (IBM SPSS Statistics for Windows, version 29.0, IBM Corp., Armonk, NY), where *p*-values < 0.05 were considered statistically significant. The normality of the variables was assessed using the Kolmogorov–Smirnov test. As the variables did not follow a normal distribution, non-parametric tests were applied. Data were presented using median and interquartile range for continuous variables, and frequency and percentage for categorical variables (participant characteristics were described using mean and standard deviation). The Friedman test was applied to examine the evolution of parameters across the MC. Effect size for the Friedman test was estimated using Kendall's coefficient of concordance (W), interpreted as small (W = 0.10), moderate (W = 0.30), and strong (W ≥ 0.50) agreement (34). *post hoc* comparisons were performed using Bonferroni-adjusted pairwise tests. The significance of the findings was further assessed by calculating the effect size using Cohen's d, as outlined below (35): d < 0.1 indicates a very small effect, d < 0.2 small, d < 0.5 medium, d < 0.8 large, d < 1.2 very large, and d > 2.0 huge (36).

## 3 Results

Participants’ baseline characteristics are described in Table 1. Seven participants were excluded from further analysis due to serum progesterone levels below 16 nmol·L⁻^1^ in MLP. The final study sample comprised *n* = 20.

### 3.1 Participant characteristics and hormonal verification

Endogenous sex hormone concentrations and body mass were evaluated across the three testing days. Progesterone, estradiol, FSH, and LH differed significantly between test moments, confirming the expected hormonal fluctuations across menstrual cycle phases. Body mass also exhibited small but statistically significant differences between tests.

### 3.2 Physical activity intensity and energy consumption

**Figure 2:**
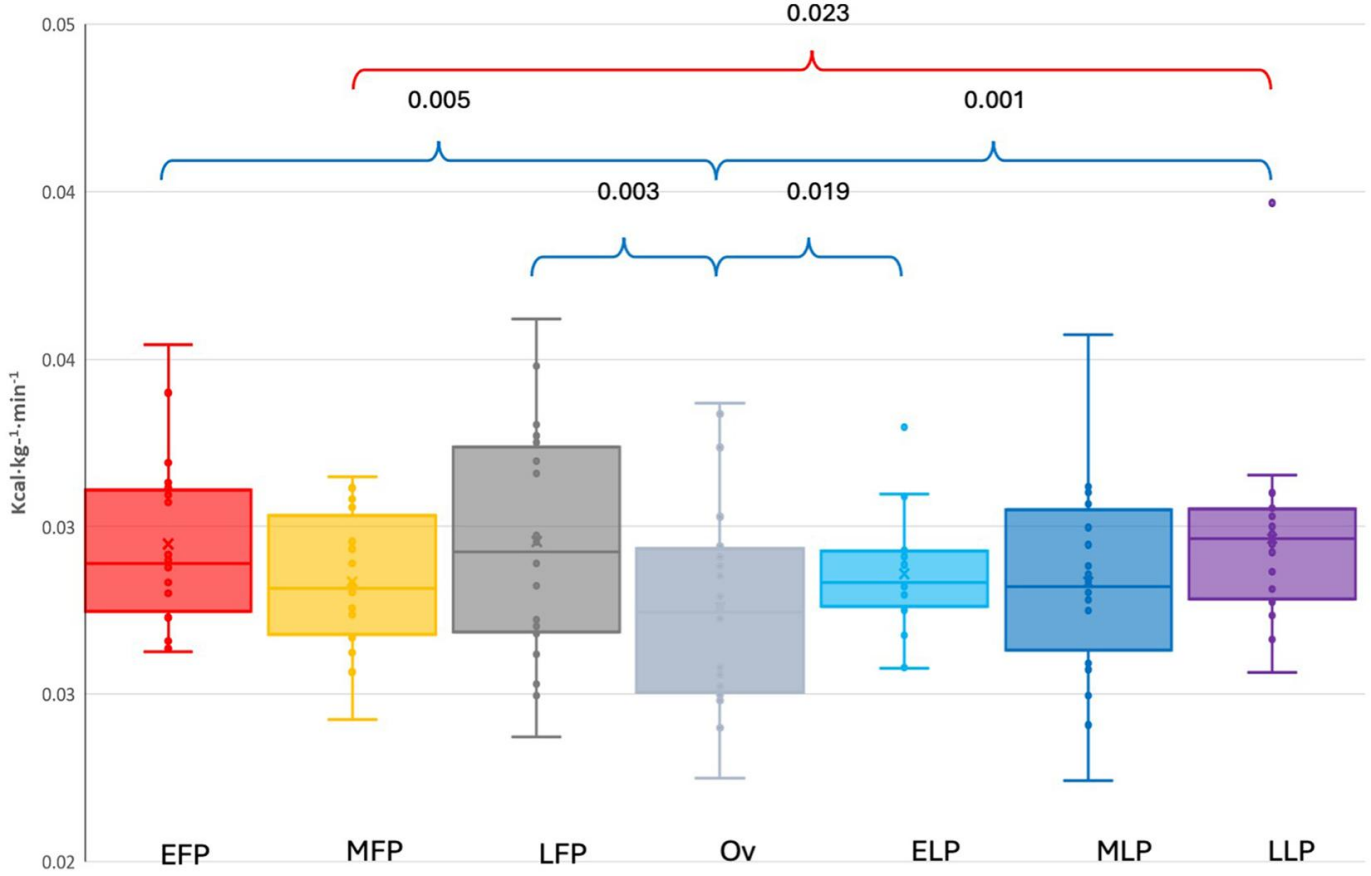
Energy consumption (kcal·kg⁻^1^·min⁻^1^) across the seven menstrual cycle phases. Values are presented as median and interquartile range of kcal·kg⁻^1^·min⁻^1^ in the different menstrual cycle phases.

Table 2 shows the variables related to physical activity intensity and energy consumption. Significant differences between the seven menstrual cycle phases were found in the percentages of vigorous, very vigorous and extremely vigorous physical activity intensities (*p* = 0.004, W = 0.158; *p* = 0.001, W = 0.185; and *p* = 0.001, W = 0.181; respectively), whereas no significant differences were observed for sedentary, light or moderate intensities (*p* = 0.111, *p* = 0.100 and *p* = 0.350, respectively). Energy consumption (kcal·kg⁻^1^·min⁻^1^) also differed across phases (*p* = 0.009, W = 0.142). Pairwise comparisons (Figure 2) showed that energy consumption was significantly lower in the ovulatory phase than in the EFP, LFP, ELP and LLP phases (Ov vs. EFP *p* = 0.005; Ov vs. LFP *p* = 0.003; Ov vs. ELP *p* = 0.019; Ov vs. LLP *p* = 0.001), and higher in LLP compared with MFP (*p* = 0.023).

**Table 2:** Physical activity intensity and energy consumption.

PA variables	*P* value	7 Menstrual cycle phases						
		EFP	MFP	LFP	Ov	ELP	MLP	LLP
Total PA(min)*		5,760 (4,320–7,200)	12,240 (10,800–14,760)	1,440	1,440	4,320	4,320	7,920 (7,920–10,800)
Total Kcal		11,407 (8,396–13,185)	22,993 (18,360–26,150)	2,686 (2,271–3,023)	2,516 (2,024–3,014)	7,762 (6,723–8,487)	7,824 (6,618–8,554)	14,890 (12,593–19,941)
Kcal·kg^−1^·min^−1^	**0**.**009**	0.029 (0.027–0.031)^d^	0.028 (0.027–0.030)^g^	0.029 (0.027–0.032)^d^	0.027 (0.025–0.029)^aceg^	0.028 (0.028–0.029)7^d^	0.028 (0.026–0.030)	0.030 (0.028–0.031)^b.d^
PA Intensity:		% min	% min	% min	% min	% min	% min	% min
Sedentary	0.111	71.09 (58.76–74.93)	72.29 (69.30–76.62)	71.01 (63.33–77.43)	74.51 (65.35–81.91)	71.63 (69.33–75.32)	72.22 (64.02–78.36)	69.11 (63.76–73.70)
Light	0.100	26.00 (22.62–37.03)	24.02 (21.50–28.54)	24.20 (20.14–32.99)	22.71 (16.68–32.95)	24.61 (21.96–28.86)	24.79 (20.69–32.30)	26.22 (24.69–32.33)
Moderate	0.350	2.26 (1.25–2.91)	2.05 (1.15–2.91)	2.15 (1.08–3.99)	1.25 (0.38–2.29)	1.79 (1.45–2.65)	2.36 (1.00–3.04)	2.54 (1.39–3.31)
Vigorous	**0**.**004**	0.07 (0.03–0.25)^d^	0.09 (0.03–0.23)^d^	0.07 (0.00–0.35)^d^	0.00 (0.00–0.07)^abcefg^	0.06 (0.02–0.19)^d^	0.09 (0.00–0.26)^d^	0.08 (0.03–0.18)^d^
Very Vigorous	**0**.**001**	0.26 (0.06–0.55)^d^	0.18 (0.09–0.35)^d^	0.03 (0.00–0.28)^d^	0.00 (0.00–0.12)^abcefg^	0.14 (0.05–0.54)^d^	0.19 (0.01–0.32)^d^	0.26 (0.07–0.45)^d^
Extremely Vigorous	**0**.**001**	0.00 (0.00–0.11)^e^	0.01 (0.00–0.15)^d^	0.00 (0.00–0.14)^d^	0.00 (0.00–0.00)^befg^	0.10 (0.01–0.19)^acd^	0.02 (0.00–0.19)^d^	0.06 (0.01–0.11)^d^

EFP, Early Follicular Phase; MFP, Mid-Follicular Phase; LFP, Late Follicular Phase; Ov, Ovulatory Phase; ELP, Early Luteal Phase; MLP, Mid-Luteal Phase; LLP, Late Luteal Phase.

Values are presented as median and interquartile range. Medians with different superscript letters indicate significant differences (*p* < 0.05): ^a^ differences with EFP; ^b^ differences with MFP; ^c^ differences with LFP; ^d^ differences with Ov; ^e^ differences with ELP; ^f^ differences with MLP; ^g^ differences with LLP.

Bold type indicates significant differences (*p* < 0.05).

* Total Phase minutes.

**Figure 3:**
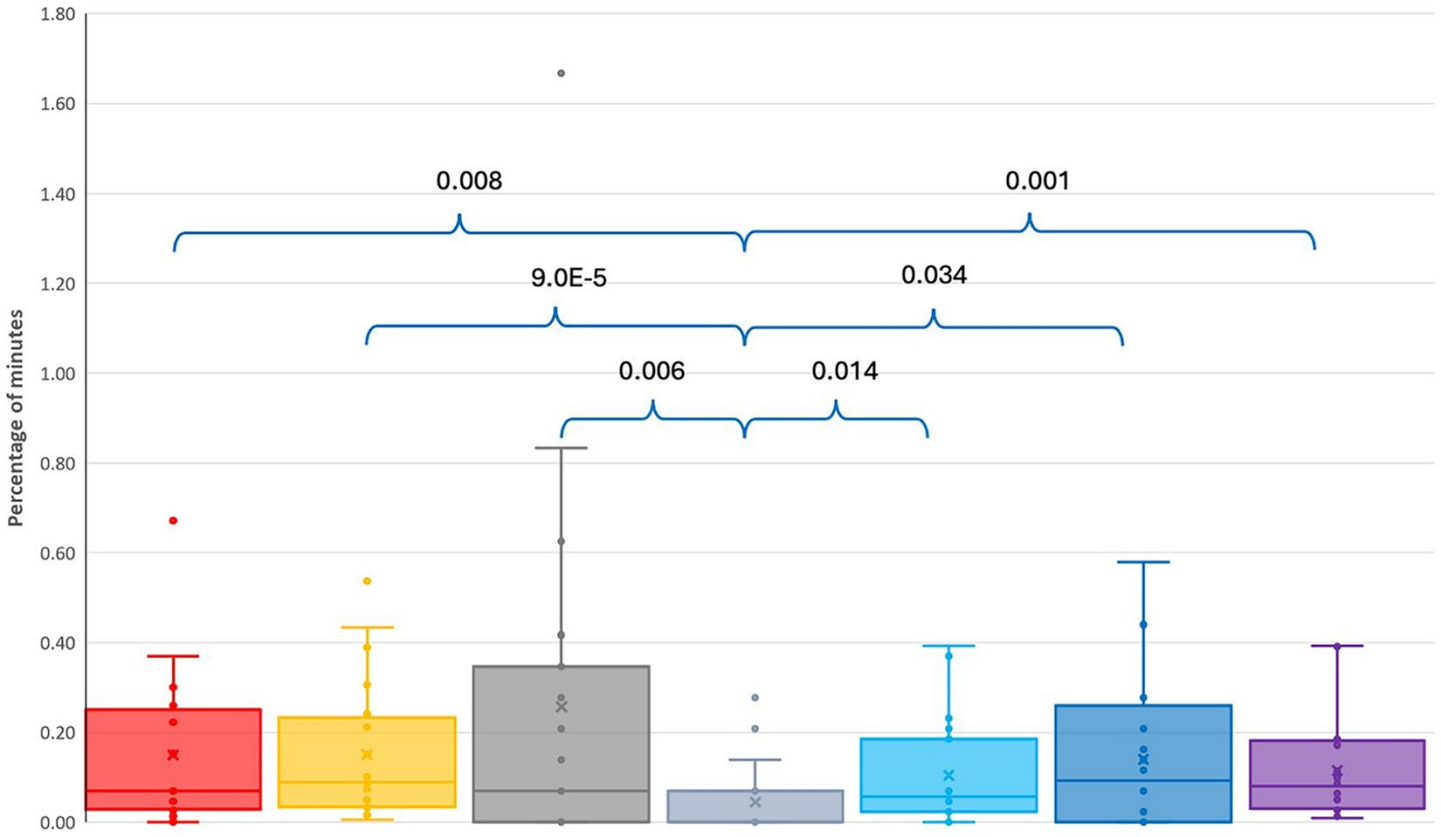
Percentage of time spent in vigorous physical activity across the seven menstrual cycle phases. Values are presented as median and interquartile range of the percentage of time spent in vigorous physical activity in the different menstrual cycle phases.

Significant differences in vigorous intensity physical activity were found between the ovulatory phase and all other six phases of the menstrual cycle (Ov vs. EFP *p* = 0.008; Ov vs. MFP *p* < 0.001; Ov vs. LFP *p* = 0.006; Ov vs. ELP *p* = 0.014; Ov vs. MLP *p* = 0.034; Ov vs. LLP *p* = 0.001), with the lowest values observed in the ovulatory phase (Figure 3).

**Figure 4:**
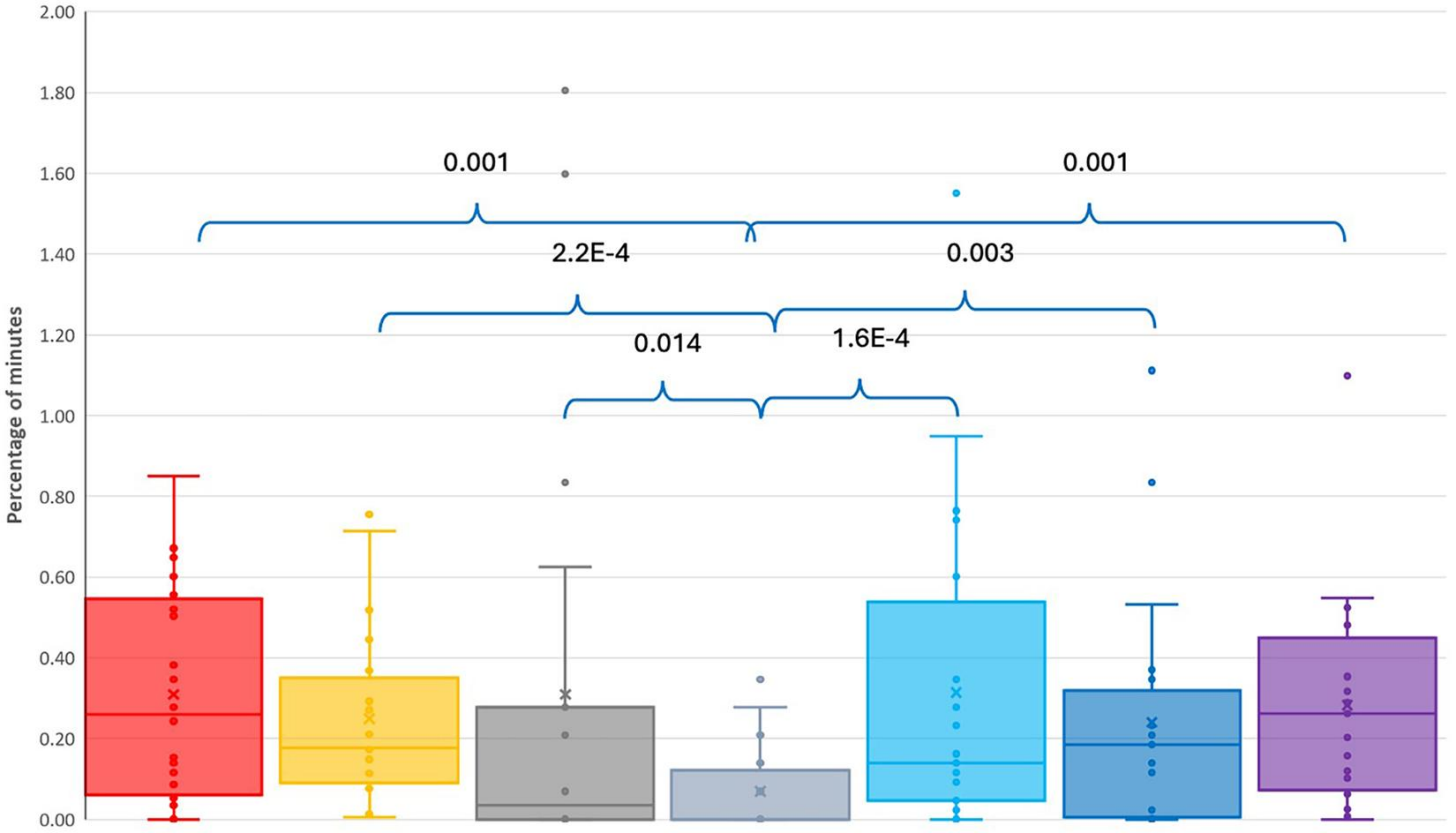
Percentage of time spent in very vigorous physical activity across the seven menstrual cycle phases. Values are presented as median and interquartile range of the percentage of time spent in very vigorous physical activity in the different menstrual cycle phases.

Significant differences in very vigorous intensity physical activity were found between the ovulatory phase and all other six phases of the menstrual cycle (Ov vs. EFP *p* = 0.001; Ov vs. MFP *p* < 0.001; Ov vs. LFP *p* = 0.014; Ov vs. ELP *p* < 0.001; Ov vs. MLP *p* = 0.003; Ov vs. LLP *p* = 0.001), with the lowest values observed in the ovulatory phase (Figure 4).

**Figure 5:**
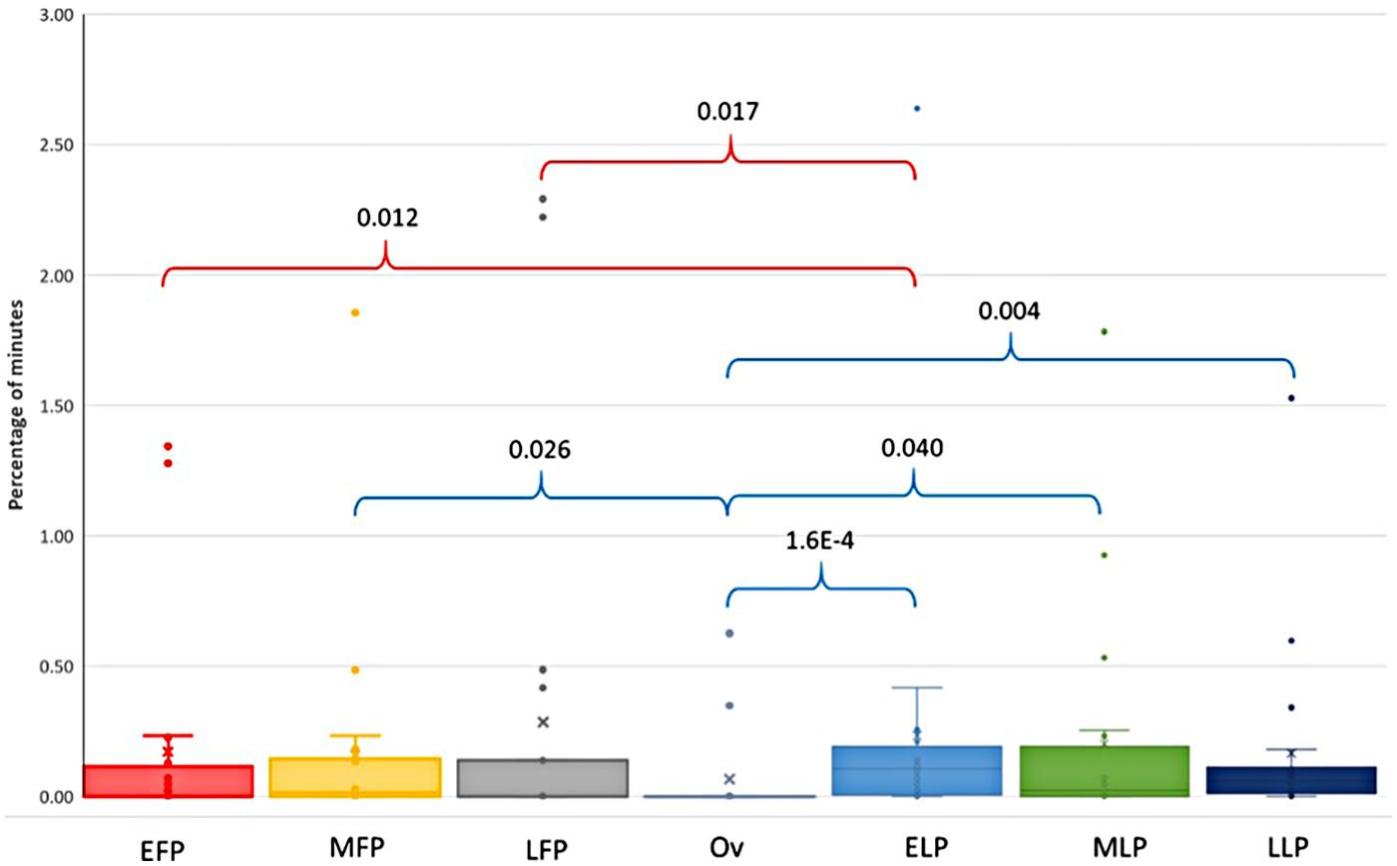
Percentage of time spent in extremely vigorous physical activity across the seven menstrual cycle phases. Values are presented as median and interquartile range of the percentage of time spent in extremely vigorous physical activity in the different menstrual cycle phases.

Significant differences in extremely vigorous intensity physical activity were found between the ovulatory phase and the LFP, ELP, MLP and LLP phases of the menstrual cycle (Ov vs. LFP *p* = 0.026; Ov vs. ELP *p* < 0.001; Ov vs. MLP *p* = 0.040; Ov vs. LLP *p* = 0.004), with the lowest values observed in the ovulatory phase (Figure 5). Differences were also observed between EFP and ELP (*p* = 0.012) and between LFP and ELP (*p* = 0.017).

## 4 Discussion

The primary finding of this study is that in healthy, eumenorrheic athletes, physical activity performed at vigorous, very vigorous, and extremely vigorous intensities, along with energy expenditure, varies across the menstrual cycle. Specifically, the lowest values in these high-intensity domains were consistently observed during the ovulatory phase. In contrast, sedentary, light, and moderate activities remained stable, suggesting that while overall activity volume is largely maintained, the intensity profile appears to be more sensitive to the different phases of the cycle.

However, these changes do not seem to translate into large behavioural disruptions. In the present sample, the overall percentage of time spent in daily physical activity and the lower-intensity domains did not differ between phases, and previous work has shown that training routines and batteries of strength and flexibility tests can remain largely stable despite phase-dependent differences in $\dot{V}$ O_2_max and pain sensitivity (20). This stability in overall volume is complemented by our statistical findings in high-intensity domains; although the effect sizes for energy consumption (*p* = 0.009, W = 0.142), vigorous (*p* = 0.004, W = 0.158), very vigorous (*p* = 0.001, W = 0.185), and extremely vigorous activity (*p* = 0.001, W = 0.181) are classified as small (34), their consistent statistical significance despite a reduced sample size (n) suggests a robust underlying phenomenon. This apparent dissociation is consistent with the idea that competitive and well-trained women often maintain their scheduled training and daily routines despite cyclical variation in symptoms, discomfort, or transient changes in physical fitness (37). Thus, external demands such as training schedules and competition calendars may buffer the impact of menstrual-cycle-related experiences on global activity volume, while the most demanding intensity brackets remain more sensitive to short-term fluctuations in perceived readiness, pain, fatigue, or comfort (20).

These results nuance the hypothesis that ovulation would be a “favourable window” for performance based solely on hormonal arguments (high estrogen, relatively low progesterone) (1, 2). In line with Dam et al. (38), who did not confirm greater strength during ovulation despite hormonal fluctuations, and Velten et al. (39) whose findings suggest that strength training responses and muscle fatigue are not substantially altered by menstrual cycle hormonal variation in trained women, our data showed that high-intensity daily activity and energy expenditure were actually lowest during the ovulatory phase. Similarly, several studies in female athletes have reported small or inconsistent effects of menstrual phase on performance outcomes (9, 10) especially when methodological quality is high (37, 40). Taken together, these findings support the view that menstrual cycle phase alone should not be used deterministically to predict performance or prescribe training loads. Instead, phase should be considered alongside other factors, including symptom burden, recovery status, training context, and psychological state.

To contextualise the present findings, it is important to consider how symptoms typically fluctuate across menstrual cycle phases. Previous research has shown that the early follicular and late luteal phases are usually associated with the greatest symptom burden, including dysmenorrhea, pelvic and lower back pain, bloating, fatigue, mood disturbance, irritability, and reduced subjective readiness to train (41, 42). In contrast, daily symptom tracking in eumenorrheic women using a seven-phase model has shown that the ovulatory, early luteal, and midluteal phases are, at the group level, among the most stable phases, with lower overall symptom expression (6). Nevertheless, the periovulatory transition may still involve transient symptoms in some women, such as mid-cycle abdominal pain or intermenstrual discomfort, which could temporarily influence willingness to perform high-intensity effort. Therefore, the lower vigorous and very vigorous activity observed here during ovulation should not be interpreted as evidence of a direct endocrine effect *per se*, but rather as a pattern that may reflect short-lasting physical discomfort, subjective readiness, or behavioural self-regulation.

From a clinical and applied standpoint, the pattern observed in eumenorrheic athlete female suggests that the menstrual cycle *per se* does not necessarily impair the ability to sustain daily activity, but it can modulate the “quality” or intensity profile of that activity and some key physiological capacities. Women with eumenorrheic cycles have been shown to present significant phase-related fluctuations in cardiorespiratory fitness and pain thresholds (20), and the present findings indicate that specific changes also occur in the highest physical activity intensities and in energy consumption, while overall routines are maintained. Rather than supporting rigid adaptations of training to fixed calendar days of the cycle, the accumulated evidence favours a more nuanced approach, where each athlete's symptoms, pain thresholds, $\dot{V}$ O_2_max or performance tests and objective physical activity patterns are monitored across several cycles, and individualised adjustments are implemented when relevant (19, 25).

Furthermore, the present accelerometry-based study opens interesting avenues for future research in different female profiles. Our participants were athlete women, accustomed to training demands and with verified eumenorrheic cycles. It would be valuable to test whether women with lower fitness levels, less structured training, different contraceptive patterns, or more severe symptomatology exhibit greater variability in daily physical activity and energy consumption across the cycle. Previous work has shown that hormonal contraception modifies hormonal profiles and may alter some fitness variables (20, 43, 44), and that ovulatory disturbances are relatively common in exercising women (21, 45). Future studies should ideally combine hormonal verification, objective physical activity monitoring, daily symptom tracking, and performance testing in order to clarify whether reductions in high-intensity activity are more closely related to endocrine variation, symptom burden, or behavioural adaptation.

Our findings are also broadly consistent with the framework proposed by Bruinvels et al. (19) who emphasised that many athletes continue to train and compete throughout the menstrual cycle despite cyclical symptoms. In this context, the stability observed in total PA volume in our sample may reflect the structured nature of athletic routines and the external demands of sport participation. However, our data also suggest that maintaining overall activity does not necessarily mean that all intensity domains are unaffected. Female athletes may preserve training adherence across the cycle while still showing subtle phase-related variation in the most demanding activity intensities, potentially influenced by pain, fatigue, discomfort, mood, or self-regulation of effort.

Finally, this work contributes to the current paradigm shift in sports science, moving from ignoring or excluding female physiology to integrating it systematically (13). Our findings underline that the menstrual cycle should neither be trivialised nor considered an insurmountable barrier to training and performance. Instead, it should be understood as a physiological rhythm that interacts with symptomatology, recovery processes, psychological state, training demands, and daily behaviour. Incorporating systematic menstrual cycle monitoring, along with objective measures such as accelerometry and performance tests, into routine athlete support can facilitate more personalised, evidence-based training strategies, potentially improving both performance and long-term health in women.

Overall, the literature remains heterogeneous and, at times, contradictory, which confirms that this is still an evolving field with important unresolved questions. More rigorous longitudinal studies using precise phase verification and concurrent assessment of symptoms, behaviour, and performance are needed to better explain how menstrual cycle experiences influence high-intensity physical activity in female athletes.

### 4.1 Strengths and limitations

From a methodological perspective, this study addresses several limitations repeatedly highlighted in the literature on women and the menstrual cycle (12, 13). A major strength is the rigorous hormonal verification of menstrual cycle status. Only eumenorrheic cycles were included, and ovulation was confirmed using a combined strategy of menstrual cycle mapping, urinary LH detection, and mid-luteal serum progesterone concentrations ≥16 nmol·L⁻^1^, following criteria applied in previous work (20) and other high-quality studies (7, 43). This approach minimized the risk of misclassifying anovulatory cycles or luteal phase deficiency, a common source of bias in earlier research (21, 46). In addition, the use of a seven-phase model, adapted from Recacha-Ponce et al. (6), provides a more granular and standardized characterization of the menstrual cycle than traditional three-phase schemes, facilitating comparisons across studies and contributing to ongoing efforts to reduce ambiguity in menstrual cycle phase classification and nomenclature.

Furthermore, a notable strength of this research is the objective quantification of daily physical activity through continuous wrist-worn accelerometry (GENEActiv). The use of this validated device (17) and the application of triaxial cut points specifically developed for active populations (29) overcomes the inherent limitations of self-reported data, such as recall bias or subjective perceptions of performance (14, 18). Ultimately, the integration of precise hormonal profiling with continuous, objective accelerometry represents a significant methodological advancement, aligning with the highest standards recently demanded for research in female athletes (13).

However, this study has limitations that should be acknowledged. First, the sample size, although comparable or larger than many previous menstrual cycle studies in athletes (1, 4, 12) remains relatively small and may limit the generalisability of the findings. The sample included both team and individual sport athletes, the final sample size (*n* = 20) limited the feasibility of a stratified analysis by sport modality. Future studies with larger cohorts should explore whether the interaction between MC and PA differs between individual and team-based disciplines. Second, participants were healthy, physically athlete female with eumenorrheic cycles and specific training characteristics; therefore, results cannot be directly extrapolated to sedentary women, those with menstrual disturbances or users of hormonal contraception. Finally, this study focused on one menstrual cycle per participant; longitudinal designs across multiple cycles would provide more robust information about intra-individual stability and the potential influence of inter-cycle variability.

## 5 Conclusions

In eumenorrheic athletes, overall daily physical activity volume remains relatively stable across the menstrual cycle; however, high-intensity activity and energy expenditure show phase-related variations, with a notable decrease observed during the ovulatory phase. These findings suggest that higher-intensity domains may be more susceptible to menstrual cycle fluctuations than total activity volume. Incorporating systematic cycle monitoring and objective assessments, such as accelerometry, could facilitate the development of evidence-informed and personalized strategies to optimize both performance and long-term health in women.

## Data Availability

The raw data supporting the conclusions of this article will be made available by the authors, without undue reservation.
